# Combining solubilization and controlled release strategies to prepare pH-sensitive solid dispersion loaded with albendazole: *in vitro* and *in vivo* studies

**DOI:** 10.3389/fvets.2024.1522856

**Published:** 2024-12-20

**Authors:** Dehai Su, Mingyang Bai, Chaoqun Wei, Xiangyang Long, Xuezhen Liu, Xiangguang Shen, Huanzhong Ding

**Affiliations:** Guangdong Key Laboratory for Veterinary Drug Development and Safety Evaluation, College of Veterinary Medicine, South China Agricultural University (SCAU), Guangzhou, China

**Keywords:** albendazole, controlled release, HPMC-AS, pharmacokinetics, solid dispersion

## Abstract

Albendazole (ABZ), classified as a class II basic drug under the Biopharmaceutics Classification System (BCS), is widely recognized for its therapeutic efficacy in treating and preventing trichuriasis. However, despite its clinical relevance, ABZ’s oral administration presents challenges due to its poor solubility and pH sensitivity, which diminish its therapeutic effectiveness. Additionally, high dosing regimens of ABZ pose risks of developmental toxicity in animal models. This study developed a pH-sensitive solid dispersion of albendazole (ABZ-pHs-SD) using Glyceryl Monostearate (GM) in conjunction with Hypromellose Acetate Succinate (HPMC-AS). Characterization via Scanning Electron Microscopy (SEM), Powder X-ray Diffraction (PXRD), Differential Scanning Calorimetry (DSC), and Fourier Transform Infrared Spectroscopy (FT-IR) confirmed the high dispersion of ABZ in a crystalline state within the carrier. Furthermore, we compared the *in vitro* dissolution profile, pharmacokinetics, and intestinal drug concentration of ABZ-pHs-SD with commercially available formulations. Our findings demonstrated that ABZ-pHs-SD exhibited an excellent dissolution profile, significantly increasing the solubility of ABZ in water by 3.15 times. The formulation effectively prevented drug release in acidic environments while maintaining a slow release in weakly alkaline conditions. Additionally, compared to commercial formulations, ABZ-pHs-SD showed significantly lower C_max_ (4.70 ± 1.16 vs. 6.83 ± 0.66 μg/mL) and higher T_max_ (5.5 ± 0.93 vs. 3.75 ± 0.71 h) *in vivo*, achieving elevated drug concentration levels in the cecal and colonic environments (*p* < 0.01) without significantly decreasing bioavailability. Overall, our research findings indicate that ABZ-pHs-SD serves as a promising drug delivery strategy for the poorly soluble and pH-sensitive ABZ. Particularly, the simple preparation of solid dispersion demonstrates strong industrial feasibility.

## Graphical abstract

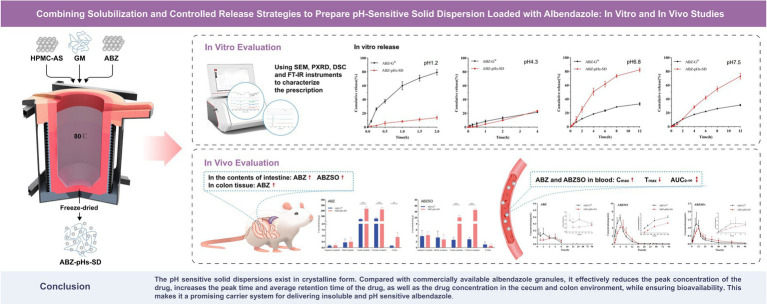

## Highlights

- **Improved solubility:** The pH-sensitive solid dispersion of albendazole with enhanced water solubility.
- **Controlled release:** The prescription effectively prevented drug release in acidic environments and facilitated slow release in weakly alkaline conditions.
- **Pharmacokinetic benefits:** Lower peak concentrations, longer peak times, and average retention times were observed in rats without significantly reducing bioavailability.
- **Targeted delivery:** Compared to commercially available formulations, after 24 hours of administration, it has a higher drug concentration in the cecum and colon environment, which is expected to improve the targeting of drug delivery.

## 1 Introduction

Trichuriasis is a zoonotic parasitic disease caused by gastrointestinal nematodes, specifically whipworms, which pose a significant threat to both human health and livestock (1). Whipworm infections affect approximately 1.049 billion people worldwide (2), resulting in substantial morbidity. The low cure rates and high reinfection rates make the treatment of whipworm infections particularly challenging (3). The life cycle of all species within the genus Trichuris is similar; upon ingestion of food or water contaminated with embryonated eggs, the eggs hatch in the large intestine (cecum and/or proximal colon) (4) and ultimately colonize and reproduce within the colon (5). Regular administration of anthelmintics, such as albendazole and mebendazole, to high-risk populations is a global strategy aimed at controlling the incidence of whipworm infections (6).

Albendazole (ABZ), a BCS II class alkaline drug (7) developed by GSK in 1975 (8), is recommended by WHO for the treatment and prevention of whipworm infections. After oral administration, ABZ undergoes a significant first-pass effect and is rapidly metabolized into albendazole sulfoxide (ABZSO) and albendazole sulfone (ABZSO_2_), with ABZ and ABZSO being the principal active components (9, 10). However, studies have shown that a single oral dose of 400 mg of ABZ provides limited clinical efficacy against whipworm infections, requiring multiple dosing, especially three dosing, to achieve desired therapeutic effects (11–13). This is likely due to ABZ’s low solubility, tendency to crystallize, and pH sensitivity, resulting in rapid dissolution and absorption in the acidic stomach environment. Meanwhile, unabsorbed drug moves into the small and large intestines–the primary habitat of gastrointestinal nematodes–where it quickly precipitates out as the pH increases (14–17), reducing effective drug concentrations at the target sites and impacting therapeutic outcomes. Moreover, certain doses of ABZ may cause developmental toxicity in animals (18); for instance, a daily dose of 10 mg/kg could induce fetal toxicity in pregnant rats and rabbits, and the same dose can lead to fetal death and teratogenic effects in pregnant food-producing animals (19).

To improve the solubility of drugs, various formulation strategies have been explored (20–22), including the use of surfactants like Tween 80 and bile salts (23), the development of self-microemulsifying formulations (24), solid lipid nanoparticles (25), solid dispersions (26–28), cyclodextrin complexes (29), and co-crystals. Amongst these, solid dispersion techniques are often preferred for enhancing the dissolution characteristics of poorly soluble drugs (30) due to their simplicity, stability, reproducibility, and scalability. However, while such strategies enhance ABZ solubility, they also seemly tend to increase the rate and peak concentrations of the drug in the bloodstream when released rapidly in the acidic stomach, potentially heightening the risk of side effects.

Targeted enteric controlled release technology offers a means to deliver pharmacologically active compounds effectively to predetermined targets within the gastrointestinal tract, thereby maximizing local therapeutic effects while minimizing side effects. Various techniques, including time-dependent delivery, pH-dependent systems, and delivery systems that utilize colon-residing bacteria or enzymes produced by these bacteria to effect drug release, have been employed for intestinal targeting (31). To date, pH-dependent systems have found practical application by exploiting the gradual increase in gastrointestinal pH from the stomach (pH 2–3) through the small intestine (pH 6.5–7) to the colon (pH 7.0–8.0) (32). These systems employ pH-sensitive materials that dissolve at specific pH levels, thereby controlling drug release. Typically, drugs need to dissolve to exert their therapeutic effects. However, if ABZ is simply encapsulated in a pH-sensitive material and delivered to the slightly alkaline environment of the intestine, its dissolution may be hindered due to its inherent low solubility and pH sensitivity. This could potentially challenge the drug’s effectiveness at the target site.

To address this challenge, this study combines the solubilization advantage of solid dispersions with enteric-targeted controlled release technology. Using Glycerol Monostearate (GM) as a poorly soluble excipient and Hypromellose Acetate Succinate (HPMC-AS) as a pH-sensitive solubilizing agent, we have prepared a pH-sensitive solid dispersion of ABZ (ABZ-pHs-SD) via the melt-extrusion method. This innovative formulation strategy aims to enhance ABZ solubility and mitigate its rapid dissolution and absorption in the gastric acidic environment, thus avoiding excessive peak plasma concentrations. At the same time, it aims to improve ABZ solubility in the weakly alkaline intestinal environment and to preliminarily validate the formulation’s targeting performance in the cecum. These findings will provide theoretical and experimental guidance for the development of novel ABZ formulations and their subsequent clinical trials in target animal models.

## 2 Materials and methods

### 2.1 Materials

Albendazole (Lot: 20210125) was supplied by Desert Pharmaceutical Co., Ltd. (Ningxia, China). Hypromellose Acetate Succinate (HPMC-AS, Lot: 220603) was purchased from Sunhere Pharmaceutical Excipients Co., Ltd. (Anhui, China). Glyceryl Monostearate (GM, Lot: 20230501) was obtained from Tianzheng Pharmaceutical Accessories Co., Ltd. (Xi’an, China). The albendazole standards (Lot: 2353168), albendazole sulfone standards (Lot: 2357608), albendazole sulfoxide standards (Lot: 2354296), and mebendazole standard (MBZ, Lot: 2204156) were all purchased from Anpel-Trace Standard Technical Services Co., Ltd. (Shanghai, China). Albendazole granules (ABZ-G^®^) were obtained from Full Woo Biotechnology Co., Ltd. (Shanghai, China). Chromatographic-grade acetonitrile and methanol were supplied by Thermo Fisher Scientific (Massachusetts, United States). All water referenced in the manuscript is ultrapure water filtered using the Milli-Q Reagent System (Millipore, Billerica, MA, United States).

### 2.2 Preparation of ABZ-pHs-SD

ABZ, GM, and HPMC-AS were weighed in a mass ratio of 25:65:10 to prepare the solid dispersion. GM was melted in a water bath, and HPMC-AS and ABZ were subsequently added. The molten mixture was stirred at 80°C until the drug was completely dispersed. The molten mixture was then poured onto a pre-cooled glass plate and frozen at −20°C overnight. After freezing, the mixture was dried in a water bath incubator for 24 h, then crushed and sieved (40–50 mesh) to obtain ABZ-pHs-SD.

### 2.3 Characterization of ABZ-pHs-SD

#### 2.3.1 SEM

The surface morphology of ABZ, ABZ-pHs-SD, GM, physical mixtures (ABZ:GM:HPMC-AS = 25:65:10) and HPMC-AS was studied using a Sigma 300 scanning electron microscope (ZEISS, Germany). Samples were fixed on aluminum stubs and coated with a layer of gold. Subsequent SEM images were obtained at an accelerating voltage of 10 kV to observe the surface morphology of the samples.

#### 2.3.2 PXRD

PXRD analysis of ABZ, ABZ-pHs-SD, physical mixtures, GM, and HPMC-AS was conducted at ambient temperature using an Empyrean instrument with Cu Kα radiation (*λ* = 1.54056 Å) and generator settings of 40 kV and 40 mA. Diffraction data were collected in the 2θ scanning range of 5–35° with a step size of 0.0084° and a counting time of 2 s per step.

#### 2.3.3 DSC

Thermal analysis of ABZ, ABZ-pHs-SD, physical mixtures, GM, and HPMC-AS was performed using a Differential Scanning Calorimeter (DSC25, United States). Samples (5.8 mg) were weighed, placed in sealed aluminum pans, and scanned at a rate of 5°C/min from 25°C to 300°C under a flow of nitrogen gas. The thermograms were recorded.

#### 2.3.4 FT-IR spectroscopy

FT-IR spectra for ABZ, ABZ-pHs-SD, physical mixtures, GM, and HPMC-AS were obtained using a PerkinElmer Frontier Fourier Transform Infrared Spectrometer. The data were collected in the range of 4,000–500 cm^−1^.

### 2.4 Solubility study of ABZ, ABZ-pHs-SD, and ABZ-G^®^

Excess amounts of ABZ, ABZ-pHs-SD, and ABZ-G^®^ were added to distilled water, while excess ABZ was also added to dissolution media at pH 1.2, 4.3, 6.8, and 7.5 to obtain supersaturated solutions. The samples were shaken at 37°C and 200 rpm for 48 h. After centrifugation, the supernatant was filtered through a 0.22 μm membrane and analyzed by HPLC. Each test was performed in triplicate.

### 2.5 *In vitro* release study

The dissolution study was conducted using Dissolution Tester 2 (paddle method, RCY-808 T, Tianjin Haiyi Da, China) in a dissolution medium containing 1,000 mL of solution: pH 1.2 hydrochloric acid, pH 4.3 acetate buffer, pH 6.8 phosphate buffer, and pH 7.5 phosphate buffer. Except for the hydrochloric acid, all other media contained 1% Tween 80. The dissolution medium temperature was maintained at 37 ± 0.5°C, and the stirring speed was set at 75 rpm. Two formulations equivalent to 4 mg of ABZ (meeting sink conditions) were added to the dissolution vessels. Samples (5 mL) were collected at 0.1, 0.25, 0.5, 1, 1.5, and 2 h; and at 0.1, 0.25, 0.5, 1, 2, and 4 h; as well as at 0.1, 0.25, 0.5, 1, 2, 4, 6, 8, and 12 h. After each collection, 5 mL of isothermal medium was immediately replenished. The collected solutions were filtered through a 0.22 μm membrane and analyzed by HPLC. Each test was performed in triplicate, with the release rate obtained from standard regression equations.

### 2.6 *In vivo* pharmacokinetic study

Male SD rats (280 ± 20 g) were fasted for 12 h with free access to water. The rats were randomly divided into two groups (*n* = 8) and received ABZ-pHs-SD or ABZ-G^®^ via oral gavage at a dose of 45 mg/kg (equivalent to ABZ). Blood samples (0.5 mL) were collected at 0.5, 1, 2, 4, 6, 8, 12, 16, 20, 24, 36, 48, 72, and 96 h post-dosing. Each blood sample was centrifuged at 4000 rpm for 10 min at 4°C to obtain plasma, which was then frozen at −20°C for further analysis.

### 2.7 Intestinal drug concentration study

Male SD rats (280 ± 20 g) were fasted for 12 h with free access to water. The rats were randomly divided into two groups (*n* = 5) and received ABZ-pHs-SD or ABZ-G^®^ via oral gavage at a dose of 12.6 mg (based on ABZ). Twenty-four hours post-dosing, the rats were euthanized, and the contents of the jejunum, ileum, cecum, and colon were collected along with ileal tissue, which were then frozen at −20°C for further analysis.

### 2.8 HPLC analysis

All sample analyses were performed using HPLC (Agilent 1,100 series) combined with a UV detector set at 295 nm. For *in vitro* studies, an Agilent Extend-C18 analytical column (250 × 4.6 mm × 5 μm) was used at a temperature of 35°C. The mobile phase consisted of methanol and water (V/V = 75:25). The flow rate was set to 1 mL/min, with an injection volume of 10 μL.

For plasma, intestinal content, and ileal tissue sample processing, 50 μL NaOH (0.4 M), 100 μL MBZ (10 μg/mL), 1.0 mL acetonitrile, and 1.0 mL ethyl acetate were sequentially added to 200 μL plasma samples, 100 mg content samples, and 100 mg ileal tissue samples. The mixture was vortexed for 3 min and centrifuged at 12,000 rpm for 10 min at 4°C; the supernatant was transferred to another centrifuge tube, and 1.0 mL of ethyl acetate was added to the first tube. The previous steps of vortexing and centrifugation were repeated, and the combined supernatants were evaporated to dryness under nitrogen at 40°C. The residue was dissolved in 200 μL of mobile phase and centrifuged at 12,000 rpm for 10 min at 4°C. The supernatant was then filtered through a 0.22 μm filter for HPLC analysis. Chromatographic separation utilized an Agilent Extend-C18 analytical column (250 × 4.6 mm × 5 μm) with isocratic elution to separate ABZ and its major metabolites. The mobile phase was a mixture of 0.02 M sodium acetate solution at pH 5.0 and acetonitrile (V/V = 65:35), with a flow rate of 1.0 mL/min at a temperature of 40°C and an injection volume of 20 μL.

### 2.9 Statistical analysis

Data were analyzed using GraphPad Prism 8.0.1 (GraphPad Prism Inc., United States) for one-way analysis of variance (ANOVA) and plotting. The main pharmacokinetic parameters were calculated using the non-compartmental model in Phoenix WinNonlin 8.4.0.6172 (Certara Inc., United States).

## 3 Results

### 3.1 Characterization study of ABZ-pHs-SD

#### 3.1.1 SEM

**Figure 1:**
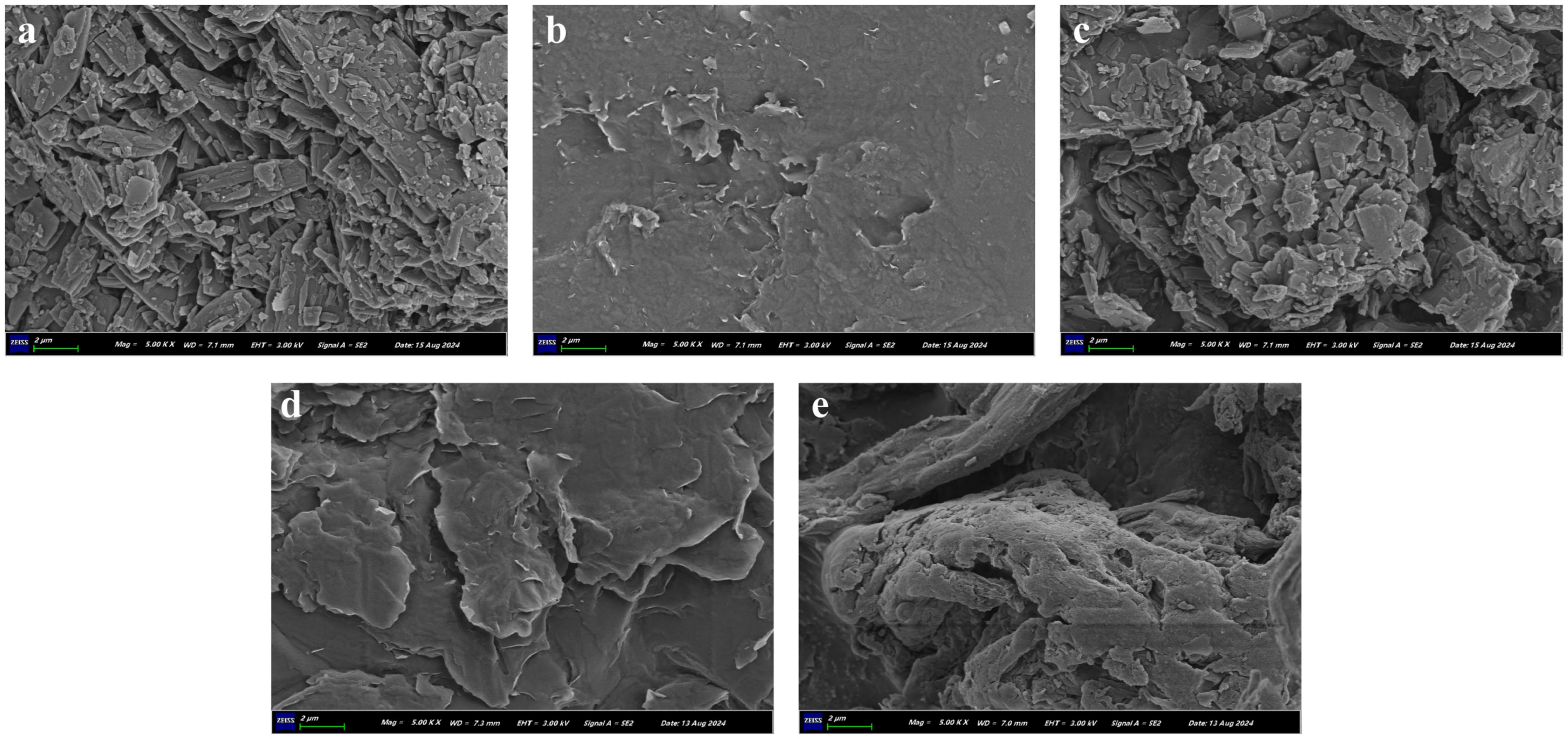
SEM images of ABZ **(A)**, ABZ-pHs-SD **(B)**, physical mixtures **(C)**, GM **(D)**, and HPMC-AS **(E)**.

We characterized ABZ, ABZ-pHs-SD, physical mixture, GM, and HPMC-AS using Scanning Electron Microscopy (SEM) to determine the morphology of the drugs within the polymer matrix (Figure 1). ABZ exhibited irregular small particles with rugged edges (Figure 1A), which was likely a primary reason for its hydrophobicity (33). Additionally, GM displayed a surface with prominent grooves, indicating a potential crystalline structure (Figure 1D), whereas HPMC-AS appeared to be dispersed in an amorphous state (Figure 1E). Although no distinct ABZ crystal structures were observed in the solid dispersion, some prominent grooves were visible (Figure 1B), likely originating from the GM. Conversely, irregular particles were still observed in the physical mixture, indicating that the crystalline structure of ABZ was not lost due to physical mixing (Figure 1C). These findings suggest that solid dispersions prepared by melting method can better disperse the drug within the polymer matrix compared to simple physical mixing.

#### 3.1.2 PXRD

**Figure 2:**
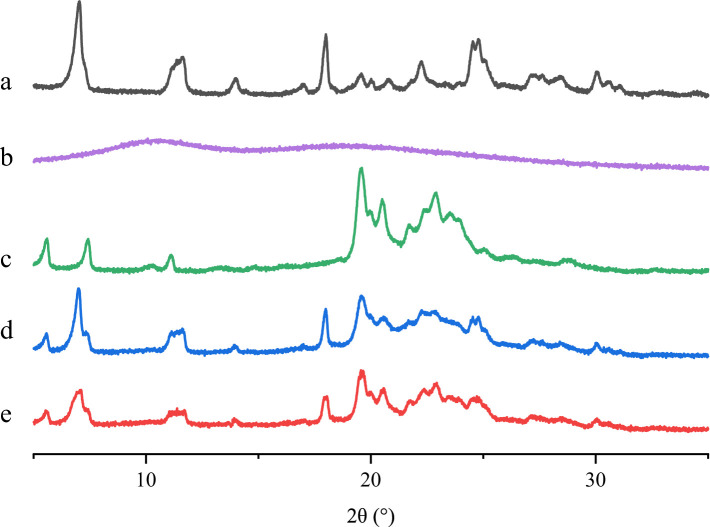
PXRD patterns of ABZ **(A)**, HPMC-AS **(B)**, GM **(C)**, physical mixtures **(D)** and ABZ-pHs-SD **(E)**.

Using Powder X-ray Diffraction (PXRD), ABZ, ABZ-pHs-SD, physical mixture, GM, and HPMC-AS were evaluated for their crystalline state (Figure 2). The original ABZ spectrum exhibited a series of high-intensity peaks at approximately 7.05°, 11.33°, 13.88°, 18.01°, 22.19°, 24.43°, and 27.20° (Figure 2A), indicating its high crystallinity, consistent with previous reports (28, 34). In contrast, the HPMC-AS spectrum showed no crystalline peaks, suggesting it was predominantly amorphous (Figure 2B). The GM spectrum displayed high-intensity peaks at approximately 5.68°, 7.44°, 11.03°, 19.66°, 22.85°, and 24.32°, indicating the crystalline structure of the carrier GM, in agreement with SEM observations. The PXRD patterns of the physical mixture and solid dispersion closely matched GM (Figures 2D,E), retaining distinct characteristic peaks, indicating that the crystalline structure of ABZ remained unaffected by its high dispersion within the polymer matrix. However, compared to the physical mixture, the solid dispersion exhibited reduced peak intensities and broadening of peak shapes in some characteristic peaks (7.05°, 11.33°, and 18.01°), suggesting a slight reduction in the crystalline structure of ABZ. This may contribute to its enhanced solubility, as amorphous forms are more readily soluble compared to crystalline forms (35).

#### 3.1.3 DSC

**Figure 3:**
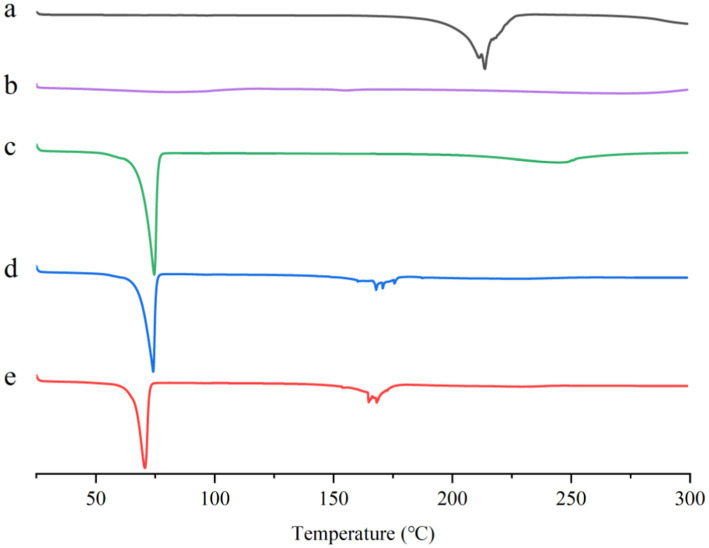
DSC curves of ABZ **(A)**, HPMC-AS **(B)**, GM **(C)**, physical mixtures **(D)** and ABZ-pHs-SD **(E)**.

We performed Differential Scanning Calorimetry (DSC) analysis on ABZ, ABZ-pHs-SD, physical mixture, GM, and HPMC-AS to determine the thermal behavior of the samples and the formation of amorphous SD, indicated by the attenuation or disappearance of drug melting peaks in the thermograms (Figure 3). The DSC thermogram of ABZ exhibited a sharp endothermic peak at 213.6°C, indicating its crystalline state (Figure 3A). HPMC-AS showed no absorption peaks, indicating the absence of crystallinity (Figure 3B). GM displayed a distinct endothermic peak at 73.4°C, consistent with its melting point (Figure 3C). The DSC curves of the physical mixture and ABZ-pHs-SD showed pronounced endothermic peaks at 73.3°C and 69.9°C, alongside weak endothermic peaks at 167.7°C and 168.0°C (Figures 3D,E). Compared to the raw material, ABZ-pHs-SD exhibited significant peak shifting and reduced intensity in the thermogram, indicating that while some crystalline forms still existed in the solid dispersion, a partial crystal transition of ABZ may have occurred. Additionally, the melting point of the solid dispersion (69.9°C) was notably lower than that of ABZ (213.6°C), GM (73.4°C), and PM (73.3°C), while the physical mixture showed no significant melting point changes. This suggests that in the physical mixture, there was no interaction between the drug and the carrier; conversely, in the solid dispersion, the drug and carrier may exist in the form of eutectic mixtures. These results align with the analysis of ABZ crystalline state in SEM and PXRD, further confirming the high dispersion of ABZ in the carrier in microcrystalline form.

#### 3.1.4 FT-IR

**Figure 4:**
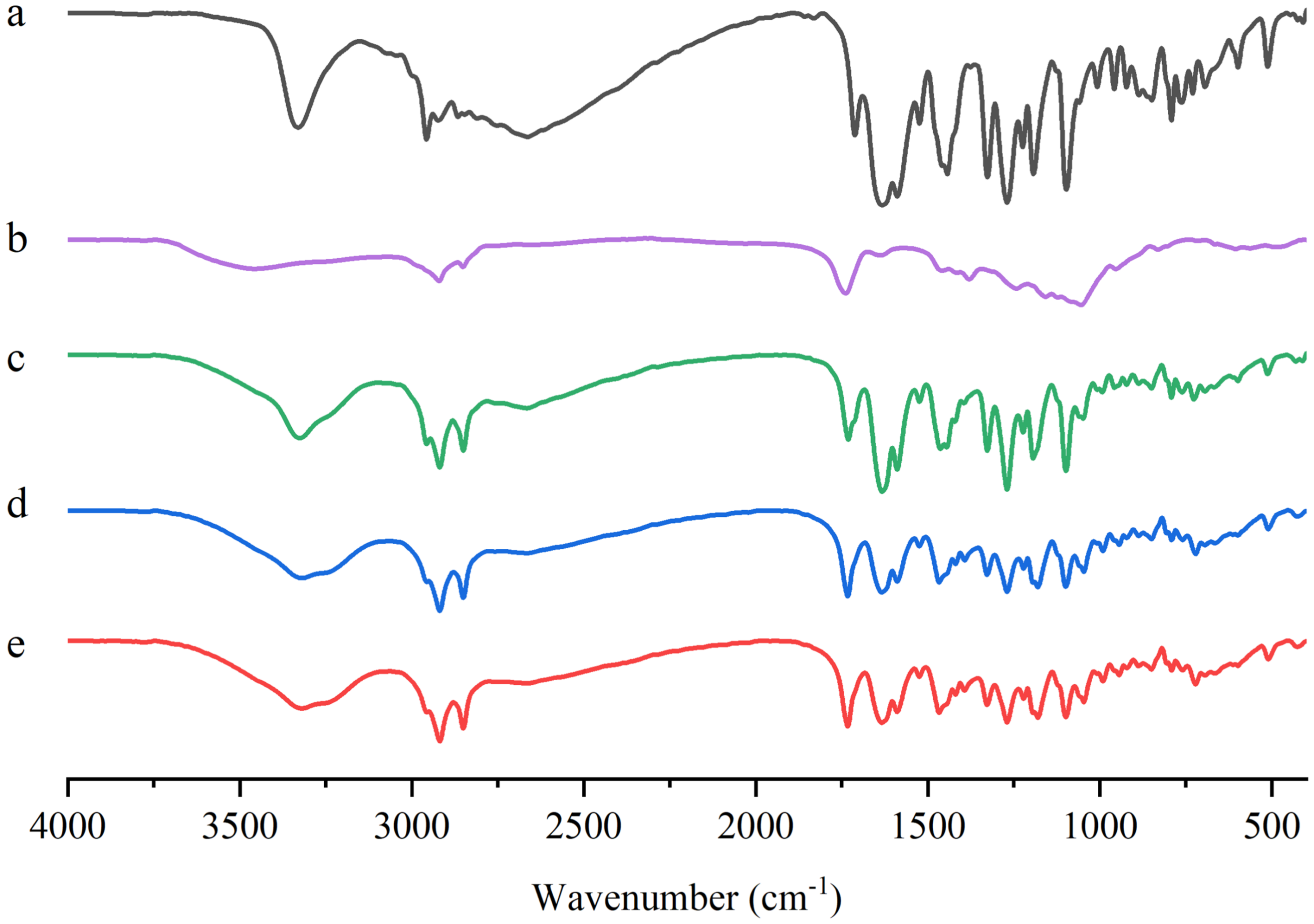
FT-IR spectra of ABZ **(A)**, HPMC-AS **(B)**, GM **(C)**, physical mixtures **(D)** and ABZ-pHs-SD **(E)**.

The Fourier Transform Infrared Spectroscopy (FT-IR) spectra of ABZ, ABZ-pHs-SD, physical mixture, GM, and HPMC-AS are shown in Figure 4. The peaks for ABZ were located at 3340 cm^−1^ (N-H stretching), 2,957 cm^−1^ (C-H stretching), 1,632 cm^−1^ (C=N stretching), 1,443 cm^−1^ (C-H bending), and 1,388 cm^−1^ (C-N stretching) (Figure 4A). The spectrum of HPMC-AS displayed peaks at 3455 cm^−1^ (O-H stretching), 1738 cm^−1^ (C=O stretching), and 1,054 cm^−1^ (C-O-C stretching) (Figure 4B). The spectrum of GM exhibited peaks at 2918 cm^−1^ (C-H stretching), 1732 cm^−1^ (C=O stretching), 1,469 cm^−1^ (C-H bending), and 1,179 cm^−1^ (C-O-C stretching) (Figure 4C). The FTIR spectrum of the physical mixture was similar to those of ABZ, GM, and HPMC-AS, indicating weak chemical interactions within the physical mixture (Figure 4D). In the FTIR spectrum of the solid dispersion, the absorption peaks of ABZ and HPMC-AS shifted from 3,340 cm^−1^ and 3,455 cm^−1^ to 3,318 cm^−1^. The broad wavenumber shift suggests the possible formation of hydrogen bonding between ABZ and the carrier HPMC-AS, which may be one of the reasons for enhancing the solubility of ABZ (36, 37).

### 3.2 Solubility study

The water solubility of ABZ, ABZ-pHs-SD, and ABZ-G^®^ was evaluated by preparing saturated solutions, as well as the solubility of ABZ in buffer media of different pH values. The initial solubility of ABZ was 0.61 ± 0.03 μg/mL, while the solubility of ABZ-G^®^ was 1.60 ± 0.10 μg/mL. However, after formulation into solid dispersions with HPMC-AS, the solubility significantly increased to 2.53 ± 0.12 μg/mL. Although ABZ could achieve a solubility of 633.50 ± 5.23 μg/mL in a pH 1.2 buffer medium, it sharply declined in weakly alkaline environments at pH 4.3, pH 6.8, and pH 7.5 to 0.69 ± 0.02 μg/mL, 0.64 ± 0.03 μg/mL, and 0.43 ± 0.01 μg/mL, respectively. This indicates that ABZ exhibits low solubility and pH sensitivity (38) (Table 1).

**Table 1:** Solubility of ABZ and different formulations in water and ABZ in different pH media.

Solvent (pH value)	Solubility (μg/mL)		
	ABZ	ABZ-G^®^	ABZ-pHs-SD
Water (7.0)	0.61 ± 0.03	1.60 ± 0.10	2.53 ± 0.12
Acid (1.2)	633.50 ± 5.23		
Buffer (4.3)	0.69 ± 0.02		
Buffer (6.8)	0.64 ± 0.03		
Buffer (7.5)	0.43 ± 0.01		

### 3.3 *In vitro* release study

**Figure 5:**
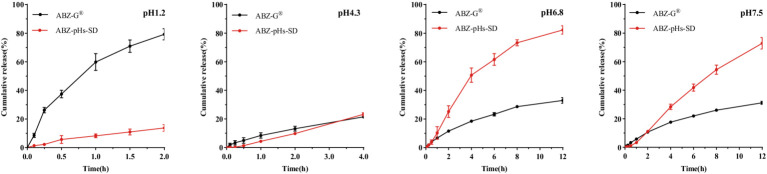
Cumulative release curves of ABZ-pHs-SD and ABZ-G^®^ in different pH media: pH 1.2, pH 4.3, pH 6.8 and pH 7.5.

In solutions of pH 1.2 and pH 4.3 (1% Tween 80), the total cumulative release of ABZ-pHs-SD was only 13.74 and 23.21%, respectively. However, in solutions of pH 6.8 and pH 7.4 (1% Tween 80), the maximum release amounts reached 82.24 and 72.85%, respectively. In contrast, the maximum release amounts of ABZ-G^®^ in these four buffer solutions were 79.18, 23.21, 32.99, and 31.20%, respectively. The results indicate that ABZ-G^®^ releases quickly and completely in strongly acidic environments, while release in weakly acidic and weakly alkaline environments is less ideal. Conversely, ABZ-pHs-SD demonstrates sufficient acid resistance while ensuring slow drug release in weakly alkaline conditions (Figure 5). This may be attributed to the properties of HPMC-AS, which exhibits good acid resistance and can inhibit the crystallization of ABZ in weakly alkaline environments (37). Additionally, the presence of GM aids in delaying drug release (39), and the combination of both components effectively controls drug release.

### 3.4 *In vivo* pharmacokinetic study

**Figure 6:**
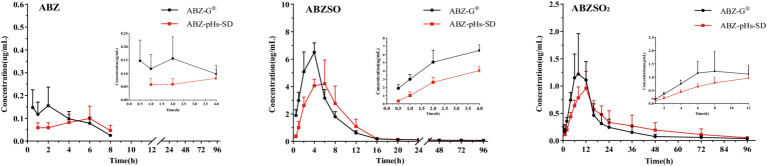
Mean plasma concentration-time curves of ABZ and its metabolites following oral administration of ABZ-pHs-SD and ABZ-G^®^ in rats (mean ± SD, *n* = 6): ABZ, ABZSO and ABZSO_2_.

The mean plasma concentration-time curves for albendazole (ABZ) and its metabolites following oral administration of ABZ-pHs-SD and ABZ-G^®^ in rats are illustrated in Figure 6, with key pharmacokinetic parameters summarized in Table 2. Notably, the levels of active components ABZ and albendazole sulfoxide (ABZSO) in the ABZ-pHs-SD group were consistently lower than those in the ABZ-G^®^ group at all time points during the first 4 hours. This finding indicates that the solid dispersion formulation effectively mitigates rapid peaks in plasma concentrations of both ABZ and ABZSO.

**Table 2:** Pharmacokinetic parameters of ABZ-pHs-SD and ABZ-G^®^ in rats.

Parameters	Unit	ABZ		ABZSO		ABZSO_2_	
		ABZ-G^®^	ABZ-pHs-SD	ABZ-G^®^	ABZ-pHs-SD	ABZ-G^®^	ABZ-pHs-SD
T_max_	h	2.00 ± 2.04	3.89 ± 2.03	3.75 ± 0.71	5.5 ± 0.93^**^	8.75 ± 2.82	11 ± 1.85
C_max_	ug/mL	0.19 ± 0.07	0.10 ± 0.05^**^	6.83 ± 0.66	4.70 ± 1.16^**^	1.47 ± 0.67	1.05 ± 0.25
t_1 /2_	h	2.18 ± 0.06	3.25	85.93 ± 32.06	97.28 ± 46.52	62.60 ± 40.08	33.82 ± 23.12
AUC_0−t_	h* ug /mL	0.65 ± 0.22	0.38 ± 0.21*	45.87 ± 3.44	41.52 ± 9.83	22.36 ± 4.13	25.50 ± 9.44
AUC_0−∞_	h* ug /mL	0.77 ± 0.21	0.55	54.26 ± 6.92	51.14 ± 14.86	25.79 ± 4.16	28.01 ± 9.38
MRT	h	2.83 ± 0.59	3.68 ± 1.30^**^	11.87 ± 0.88	14.01 ± 2.00^*^	19.93 ± 3.94	27.32 ± 5.76^**^
Ke	1/h	0.32 ± 0.01	0.21	0.009 ± 0.003	0.008 ± 0.003	0.018 ± 0.017	0.027 ± 0.015

* indicates *p* < 0.05, ** indicates *p* < 0.01 compared to ABZ-G^®^.

In terms of pharmacokinetic outcomes, ABZ-pHs-SD resulted in decreased C_max_ and increased T_max_ compared to ABZ-G^®^. Specifically, the C_max_ values for ABZ and ABZSO in the ABZ-pHs-SD group were 0.10 ± 0.05 μg/mL and 4.70 ± 1.16 μg/mL, respectively, reflecting significant reductions of 47.4 and 31.2% compared to ABZ-G^®^ (0.19 ± 0.07 μg/mL and 6.83 ± 0.66 μg/mL) (*p* < 0.01). Additionally, the T_max_ for ABZSO was significantly prolonged in the ABZ-pHs-SD group (5.5 ± 0.93 h) compared to ABZ-G^®^ (3.75 ± 0.71 h), demonstrating a delay of 1.75 h (*p* < 0.01). This effect is likely due to the robust acid resistance of glyceryl monostearate (GM) and Hypromellose Acetate Succinate (HPMC-AS), which help prevent the rapid dissolution and absorption of ABZ in the acidic environment of the stomach. Moreover, there were no statistically significant differences in the AUC_0−t_ and AUC_0−∞_ values for the active metabolite ABZSO between the two formulations, suggesting that the reduction in C_max_ does not adversely impact the bioavailability of oral ABZ while simultaneously enhancing its safety profile. This improved performance may be attributed to the solid dispersion technology and the potential hydrogen bonding interactions between HPMC-AS and ABZ, which facilitate solubilization. Furthermore, the mean residence times (MRT) for ABZ, ABZSO, and ABZSO2 in the ABZ-pHs-SD group (3.68 ± 1.30, 14.01 ± 2.00, and 27.32 ± 5.76 h, respectively) were significantly longer than those observed with ABZ-G^®^ (2.83 ± 0.59, 11.87 ± 0.88, and 19.93 ± 3.94 h) (*p* < 0.01, 0.05 and 0.01).

### 3.5 Intestinal drug concentration study

**Figure 7:**
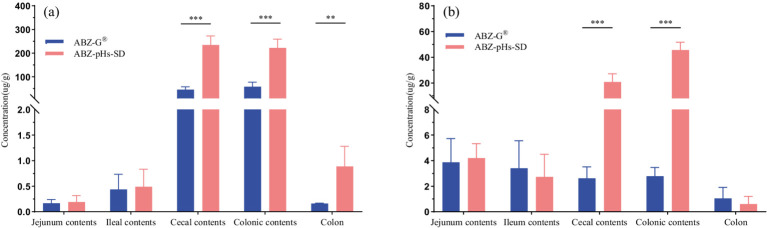
Drug concentrations of ABZ-pHs-SD and ABZ-G^®^ in intestinal contents and ileal tissue (* indicates *p* < 0.05, ** indicates *p* < 0.01, and *** indicates *p* < 0.001 compared to ABZ-G^®^).

The drug concentrations in intestinal contents and colonic tissues of rats, 24 h after oral administration of ABZ-pHs-SD and ABZ-G^®^, are presented in Figure 7. Statistically, the levels of ABZ and its metabolite albendazole sulfoxide (ABZSO) in the cecal contents for the ABZ-pHs-SD group (234.32 ± 38.47 and 20.80 ± 6.42 μg/g, respectively) were significantly higher than those in the ABZ-G^®^ group (45.46 ± 12.10 and 2.62 ± 0.90 μg/g, respectively) (*p* < 0.001). In the colonic contents, the concentrations of ABZ and ABZSO in the ABZ-pHs-SD group (221.73 ± 37.36 and 45.66 ± 6.00 μg/g) also exceeded those observed with ABZ-G^®^ (58.11 ± 19.23 and 2.78 ± 0.70 μg/g) (*p* < 0.001). Furthermore, the ABZ concentration in colonic tissue for the ABZ-pHs-SD group (0.89 ± 0.39 μg/g) was significantly greater than that for ABZ-G^®^ (0.16 ± 0.01 μg/g) (*p* < 0.01). These findings indicate that ABZ-pHs-SD has higher drug concentration levels in the cecal and colonic environments 24 h after administration, and is significantly higher than commercially available formulations. Preliminary indications suggest the potential of this formula in delivering albendazole to the cecum and colon.

## 4 Discussion

Solid dispersion systems are the preferred method for improving the dissolution and solubility of poorly soluble drugs (40, 41). When drugs are uniformly dispersed in molecular, colloidal, or microcrystalline forms, a solid dispersion system is formed, which enhances the drug’s solubility, dissolution rate, and bioavailability (42). Based on the state of the drug embedded in the polymer matrix, solid dispersions can be classified into amorphous or crystalline types. Drugs in amorphous solid dispersions exist in a high-energy state, significantly improving their solubility. However, crystalline solid dispersions have shown advantages in terms of long-term storage stability and improved drug loading, offering stronger industrial and commercial feasibility (43). In this study, we combined the benefits of solid dispersion for solubilization and enteric-targeted controlled release. We prepared pH-sensitive solid dispersions of ABZ, GM, and HPMC-AS by the melting method. Using orthogonal design, we optimized the material ratios, drug loading, and particle size, finalizing the formulation as ABZ:GM:HPMC-AS = 25:65:10, with a particle size range of 40–50 mesh. *In vitro* characterization demonstrated that the drug was incorporated in crystalline form within the polymer. Compared to commercial particles, this formulation not only exhibited higher drug loading but also enhanced solubility, increasing threefold compared to the original ABZ. This improvement may be attributed to partial polymorphic transformation, hydrogen bond formation, and the inhibitory crystallization effect of HPMC-AS during the dissolution process, consistent with the findings of Hu et al. (35–37).

In *in vitro* release studies, pH values of 1.2, 4.3, 6.8, and 7.4 were used to simulate the gastric, duodenal, jejunal, and cecal pH environments (44). Since ABZ has low solubility in water (0.61 ± 0.03 μg/mL), to better simulate the intestinal environment and meet the sink condition, 1% Tween 80 was added to the buffer. This reduced the interfacial tension between the solution and surface, ensuring the system met the 3–5 times sink condition requirement (45). Compared to commercial particles, ABZ-pHs-SD showed good acid resistance in a pH 1.2 environment, effectively avoiding rapid release in acidic conditions, while exhibiting superior release profiles at pH 6.8 and 7.4, ensuring drug release under alkaline conditions. Notably, this release behavior differs significantly from other ABZ formulations prepared using solid dispersion techniques (26, 28, 33), as well as other strategies aimed at improving ABZ’s solubility (29, 46). The mechanism controlling drug release is primarily dependent on the steric hindrance provided by GM (39) and the pH-responsive behavior of HPMC-AS. Due to its amphiphilic nature, HPMC-AS is hydrophobic under acidic conditions, working with GM to create steric hindrance that prevents drug release. However, when the pH exceeds 6.0, HPMC-AS switches to a hydrophilic state and acts as a pore-forming agent, promoting drug release from the pores. This behavior was preliminarily corroborated by pharmacokinetic and intestinal drug concentration studies in rats.

**Figure 8:**
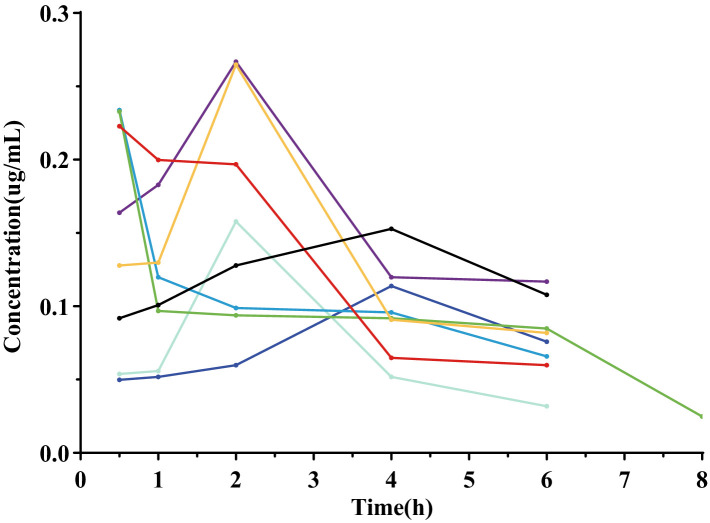
ABZ drug time curve of each rat in the commercially available pellet group.

In the pharmacokinetic study, ABZ-pHs-SD exhibited lower Cmax and higher Tmax compared to ABZ-G^®^, without significantly reducing bioavailability. The active metabolites, ABZ and ABZSO, were consistently lower at all time points within the first 4 h compared to the commercial formulation. This may be related to the solid dispersion’s excellent acid resistance observed in *in vitro* dissolution experiments. By avoiding rapid dissolution in gastric acid, the formulation mitigated fast absorption and improved the ABZSO pharmacokinetic profile. Interestingly, the average concentration-time curve for ABZ in rats showed two peak phenomenon. However, individual rat concentration-time curves (Figure 8) did not exhibit a two peak pattern, suggesting that the observed two peak nature in the average curve may result from difference in blood drug concentrations at different time points in different rats. These differences could stem from the strong first-pass effect in the gastrointestinal tract and liver, where ABZ undergoes sulfation to form ABZSO (47, 48). Variability in gastrointestinal transit, absorption rates, and liver metabolism contributes to differences in ABZ absorption and conversion, leading to differences in plasma concentrations. In addition, we found that compared to commercially available particles, the blood concentration level of ABZSO in ABZ pHs SD was slightly reduced (not statistically significant), but its concentration level in the cecum and colon environment increased significantly at 24 h after administration. This may indicate that part of the mechanism by which ABZSO kills whipworms may come from the conversion of ABZ to ABZSO in the liver, and ABZSO is excreted through bile (49) to the cecum (the target site of parasitic whipworm adults), which may be one of the potential mechanisms by which elevated blood concentration levels improve the efficacy of whipworms gastrointestinal parasites. And another part may come from the *in vitro* sulfuric acid oxidation of ABZ by intestinal microsomes, converting ABZ which was released and dissolved in the intestine and not absorbed into ABZSO. The significant increase in the concentration levels of ABZSO and ABZ in the cecum and colon environments indicates that our formula’s *in vivo* release is similar to the release performance simulated in the cecum environment in vitro release experiments, and the drug may be effectively delivered to the target site in vivo. Compared to promoting rapid dissolution and absorption of drugs through a single solubilization method, this improvement may enhance the targeting of drugs and avoid the toxic side effects of increased blood drug concentration by transporting them to the cecum through blood circulation and bile excretion. However, further research is needed to determine the effective drug concentration and actual therapeutic effect for treating whipworm disease at the target site of the target animal. Notably, although the AUC of ABZ-pHs-SD was lower in rats, the MRT values for ABZ, ABZSO, and ABZSO_2_ (3.68 ± 1.30, 14.01 ± 2.00, and 27.32 ± 5.76 h) were significantly higher than those of ABZ-G^®^ (2.83 ± 0.59, 11.87 ± 0.88, and 19.93 ± 3.94 h) (*p* < 0.01, 0.05, 0.01), suggesting that ABZ-pHs-SD has a longer residence time in vivo. This extended duration may improve the treatment and prevention of parasitic diseases, compared to the commercial formulation.

ABZ is generally not recommended for use in early pregnancy in animals, primarily because its major active metabolite, ABZSO (50), is considered teratogenic and can cross the placental barrier, detected in both the embryo and uterus (18, 51, 52). The teratogenic mechanism may involve ABZSO’s affinity for *β*-tubulin during mitosis, leading to interference with mitosis in early-stage embryos and resulting in fetal malformations. In a separate study, rats were orally administered teratogenic doses of 10.6 mg/kg or 11.24 mg/kg of ABZ or ABZSO, combined with the inhibitor SKF-525A, which prevents the oxidation of sulfur derivatives into sulfoxides. Results showed that the SKF-525A + ABZ group significantly reduced ABZ conversion to ABZSO, decreasing ABZSO exposure of blood and nearly completely inhibiting embryo toxicity, indicating a close relationship between ABZ’s embryotoxicity and ABZSO plasma concentrations (53).

Interestingly, research on ABZ formulations has primarily focused on improving its solubility and release rate to enhance bioavailability. For example, Ding Y et al. used cyclodextrin inclusion complexes to increase ABZ’s solubility and release rate in acidic environments. In pharmacokinetic studies in dogs at a dose of 25 mg/kg, the solubility of ABZ was significantly improved, with 90% of the drug released within 10 min in acid, resulting in an increase in AUC from 50.72 ± 6.28 hμg/mL to 119.95 ± 11.7 hμg/mL. However, this was accompanied by a significant increase in Cmax from 2.81 ± 0.67 μg/mL to 10.20 ± 1.91 μg/mL (29). Similarly, Castro SG et al. used P188 to prepare ABZ solid dispersions, which improved its solubility threefold, releasing 80% of ABZ in 10 min in acidic conditions. Oral administration at 25 mg/kg in mice increased AUC0-t from 13.0 ± 3.00 to 19.0 ± 4.00 h*μg/mL, with Cmax increasing from 3.50 ± 0.50 to 8.00 ± 1.00 μg/mL (54). However, it is worth noting that improving ABZ’s solubility often leads to faster release and absorption, which results in significantly higher peak blood concentrations. While this enhancement may improve systemic bioavailability and clinical efficacy, it may also exacerbate the drug’s toxic side effects, further limiting its use in pregnant animals.

To address this issue, our study introduced a controlled release strategy. By leveraging solid dispersion technology to improve ABZ’s solubility, we controlled rapid release in the gastric acid environment to avoid fast absorption, thereby reducing peak blood concentrations without significantly lowering bioavailability. This approach extends the time to reach peak concentration and prolongs the MRT. The combined effect of improved solubility and controlled release led to significant improvements in drug concentration in the cecum and colon compared to the commercial formulation. Despite promising results in *in vitro* dissolution, pharmacokinetic studies in rats, and cecal-colon distribution, further research is needed to investigate the formulation’s stability under different storage conditions, long-term stability, pharmacokinetic profiles in target animals (pigs, chickens, dogs, cats, and sheep, etc.), and its clinical efficacy and safety. Regarding intestinal concentration, the preliminary study sampled at 24 h post-administration, showing a significant increase in drug levels in the cecal and colonic environments. However, the study only provides a local and static description of intestinal distribution. To gain a more comprehensive and dynamic understanding of the formulation’s behavior in target animals’ intestines, future studies should include additional sampling time points or real-time monitoring.

## 5 Conclusion

The results from DSC, FTIR, PXRD, and SEM analyses indicated that in the ABZ-pHs-SD formulation, the drug is predominantly dispersed in the form of microcrystals within the carrier matrix. *In vitro* solubility and release studies demonstrated that ABZ-pHs-SD significantly enhanced the solubility of ABZ in water by 3.15-fold (compared to a 1.62-fold increase for ABZ-G^®^), effectively preventing drug release in both strong acid and weak acid environments while ensuring a controlled release in the weakly alkaline environment. Furthermore, pharmacokinetic studies in rats showed that, compared to ABZ-G^®^, ABZ-pHs-SD exhibited lower Cmax and higher Tmax, without significantly decreasing bioavailability. Notably, the drug concentration in the cecum and colon at 24 h post-administration was higher for ABZ-pHs-SD. These findings suggest that the pH-sensitive solid dispersion, formulated with GM and HPMC-AS, is a promising drug delivery strategy. It has the potential to enhance the solubility and targeting of albendazole while reducing peak plasma drug concentrations. This provides a solid theoretical and experimental foundation for the future development of novel ABZ formulations.

## Data Availability

The raw data supporting the conclusions of this article will be made available by the authors, without undue reservation.
